# Supplementary material for: Mode of Action of Toxin 6-Hydroxydopamine in SH-SY5Y Using NMR Metabolomics
Source: Molecules. 2025 Aug 12;30(16):3352. doi: 10.3390/molecules30163352 (PMC12388602; doi:10.3390/molecules30163352)
Supplement: Supplementary file 1 [file molecules-30-03352-s001.zip › molecules-3614223-supplementary.pdf]

Supporting Information

Mode of Action of Toxin 6-Hydroxydopamine in SH-SY5Y Using NMR Metabolomics

Roktima Tamuli [1,2], George D. Mellick [1,2], Horst Joachim Schirra [1,2,3] and Yunjiang Feng [1,2,*]

[1] Institute for Biomedicine and Glycomics, Griffith University, Nathan, QLD 4111, Australia; roktima.tamuli@griffithuni.edu.au (R.T.); g.mellick@griffith.edu.au (G.D.M.); h.schirra@griffith.edu.au (H.J.S.)
[2] School of Environment and Science, Griffith University, Nathan, QLD 4111, Australia
[3] Centre for Advanced Imaging, The University of Queensland, Brisbane, QLD 4072, Australia
* Correspondence: y.feng@griffith.edu.au

Table of Contents:

Figure S1: ^{1}H NMR spectrum of PQC endo metabolome……………………………………3

Figure S2: ^{1}H NMR spectrum of PQC endo metabolome……………………………………3

Figure S3a: Structure of pyroglutamyl alanine demonstrating ^{1}H and ^{13}C chemical shifts in ppm……………………………………………………………….…………….…...3

Figure S3b: HMBC correlations are shown using blue arrows. TOCSY correlations are shown using black bold bonds…………………………………………………….………..3

Figure S3c: STOCSY correlations using driver peak at 1.50 ppm……………………………4

Figure S4: ^{1}H and ^{1}H-TOCSY correlations of pyroglutamyl alanine……..…………………..4

Figure S5: ^{1}H and ^{13}C-HMBC correlations of pyroglutamyl alanine……..…………………..5

Figure S6: Total metabolite content of endo metabolome (left). Box-plot displaying the distribution of all datpoints (right)……………………………………………………………5

Figure S7: Total metabolite content of exo metabolome (left). Box-plot displaying the distribution of all datpoints (right)……………………………………………………………6

Figure S8: PCA bivariate loadings plot of endo metabolome of 6-OHDA treated and untreated group (new dataset)…………………………………………………………………6

Table S1: Endo metabolites identified……………………………………………………...…7

Table S2: Exo metabolites identified…………………………………………….……………8

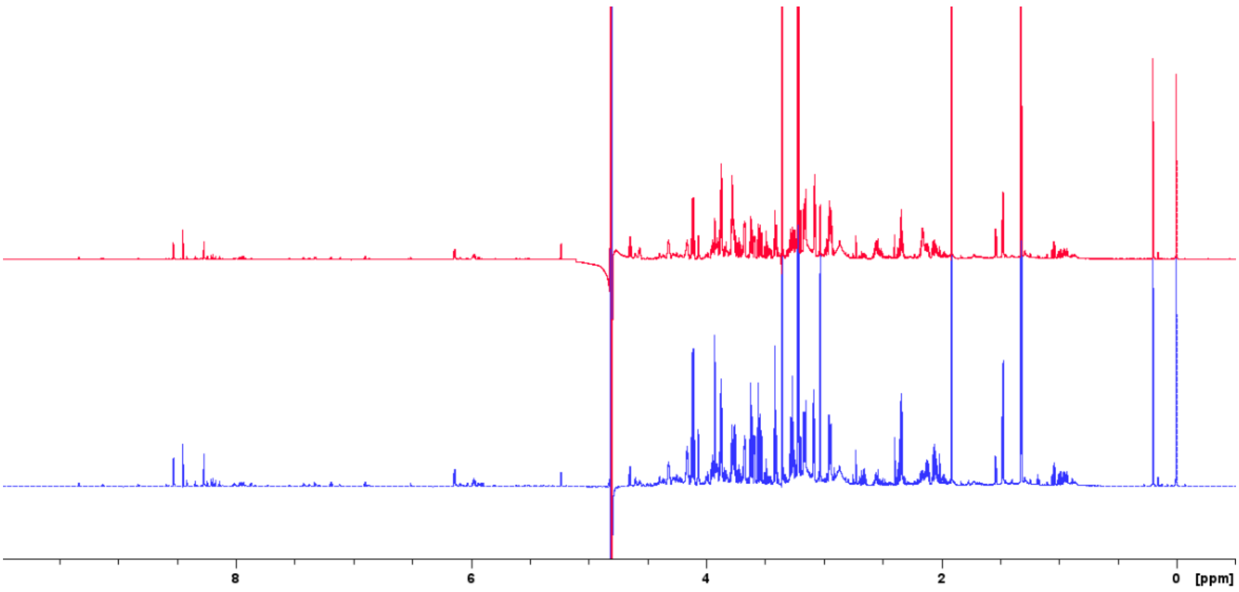

Figure S1: ^{1}H NMR spectrum of PQC endo metabolome. Blue: Untreated, Red: Treated.

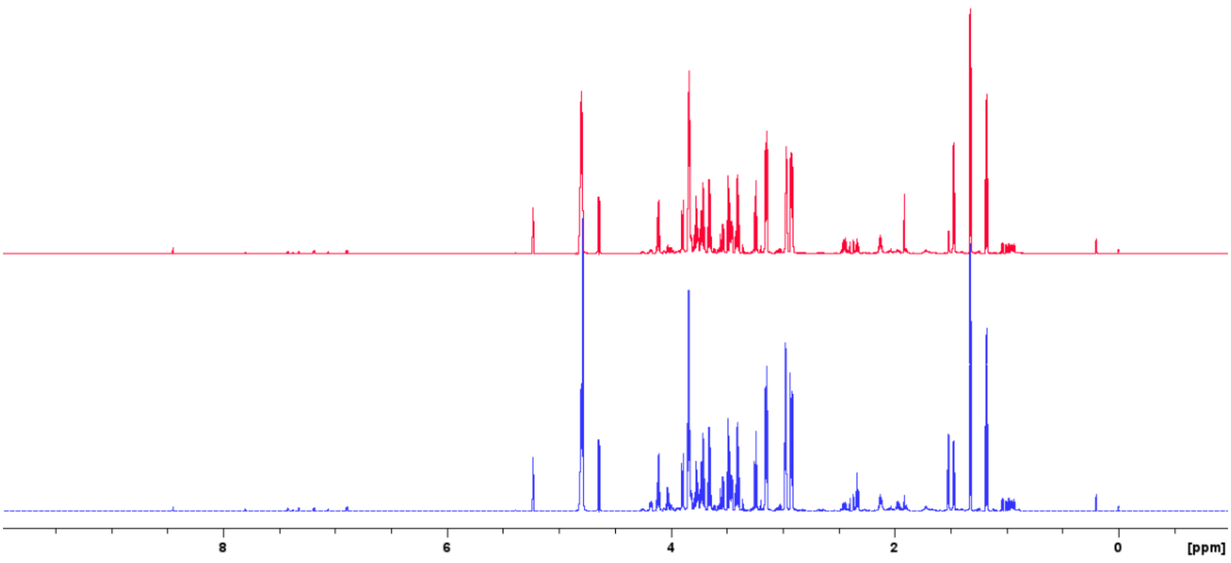

Figure S2: ^{1}H NMR spectrum of PQC exo metabolome. Blue: Untreated, Red: Treated.

a

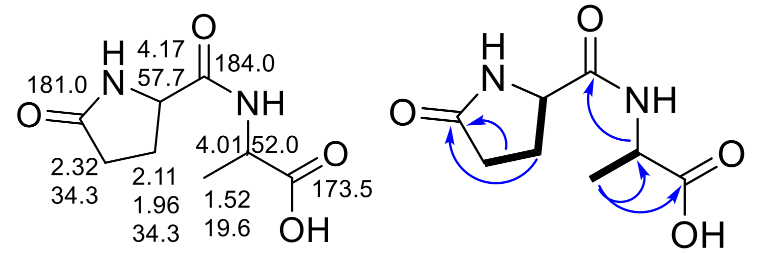

b

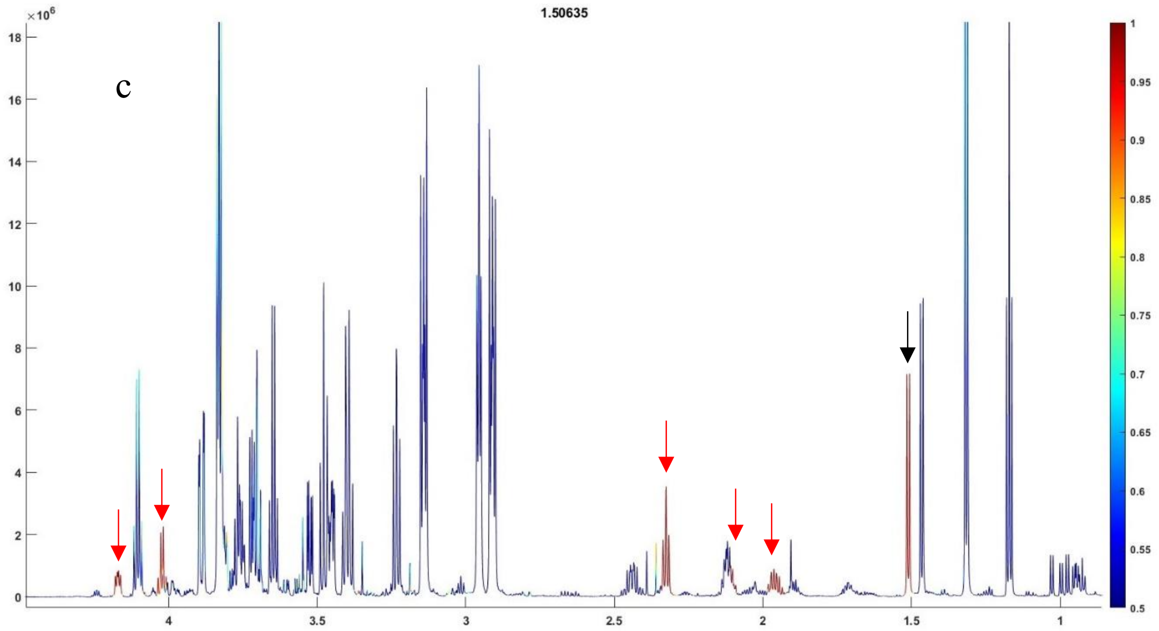

Figure S3a: Structure of pyroglutamyl alanine demonstrating ^{1}H and ^{13}C chemical shifts in ppm. S3b: HMBC correlations are shown using blue arrows. TOCSY correlations are shown using black bold bonds. S3c: STOCSY correlations using driver peak at 1.50 ppm. ^{1}H at 1.50 ppm (black arrow) correlates to ^{1}H at 1.96, 2.11, 2.32, 4.01, and 4.17 ppm (red arrow).

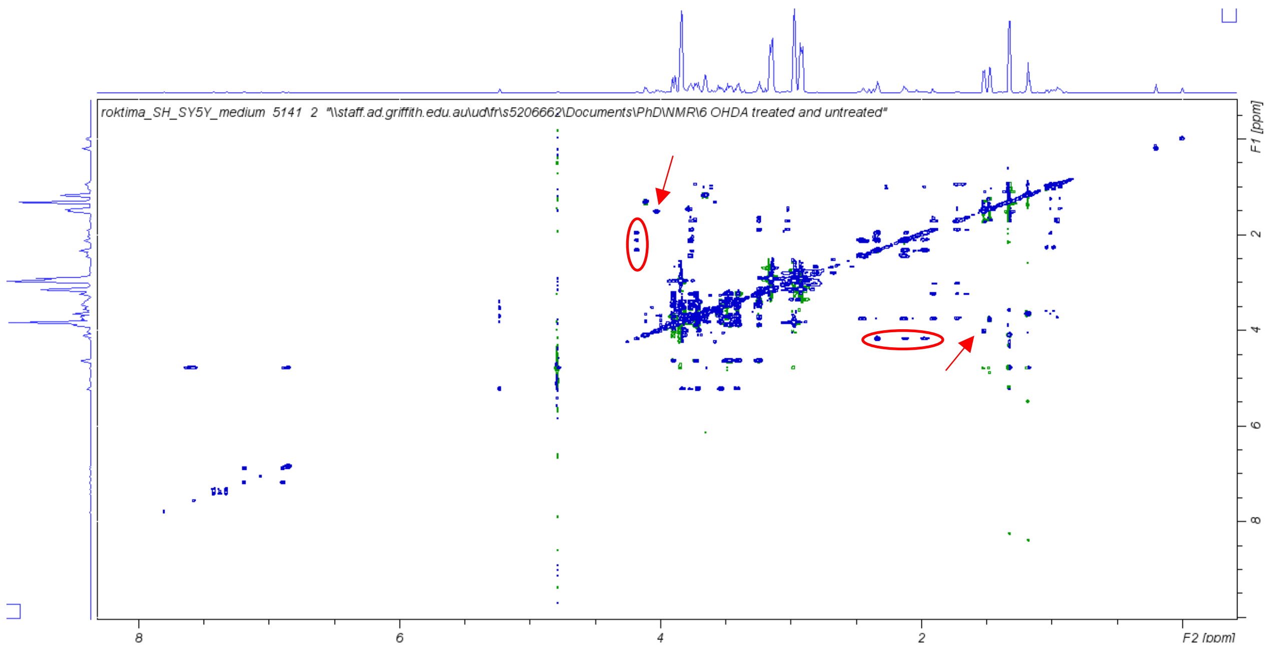

Figure S4: ^{1}H and ^{1}H-TOCSY correlations of pyroglutamyl alanine. The TOCSY correlations indicate the presence of two spin systems. ^{1}H at 4.17 ppm has TOCSY correlations with ^{1}H at 2.32, 2.11, and 1.96 ppm (red circle). ^{1}H at 1.52 ppm correlates to ^{1}H at 4.01 ppm (red arrow).

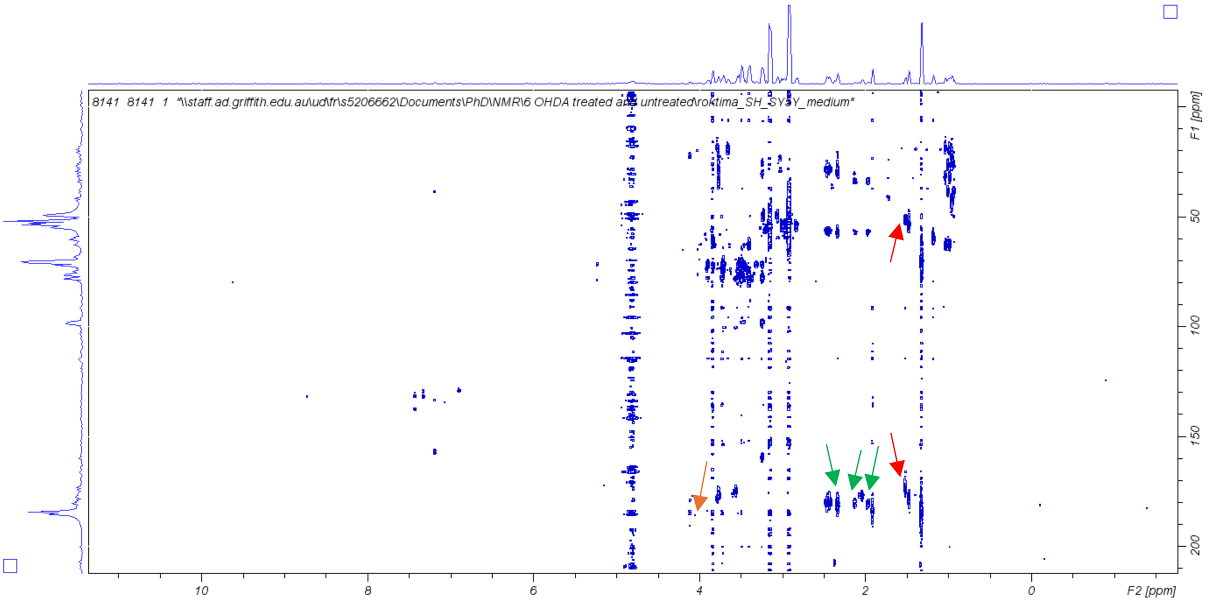

Figure S5: ^{1}H and ^{13}C-HMBC correlations of pyroglutamyl alanine. ^{1}H at 1.52 ppm correlates to carbon at ^{13}C at 52.0 and 173.5 ppm (red arrow); ^{1}H at 4.01 ppm correlates to carbon at ^{13}C at 184.0 ppm (orange arrow); ^{1}H at 1.96 ,2.11, and 2.32 ppm correlates to carbon at ^{13}C at 181.0 ppm (green arrow).

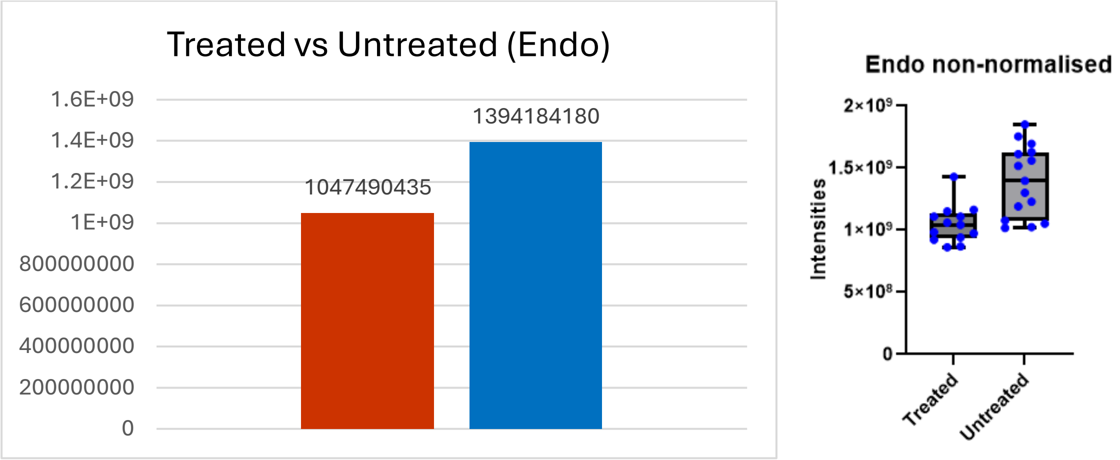

Figure S6: Total metabolite content of endo metabolome (**left**). Red: treated with 6-OHDA. Blue: untreated. Box-plot displaying the distribution of all datpoints (**right**).

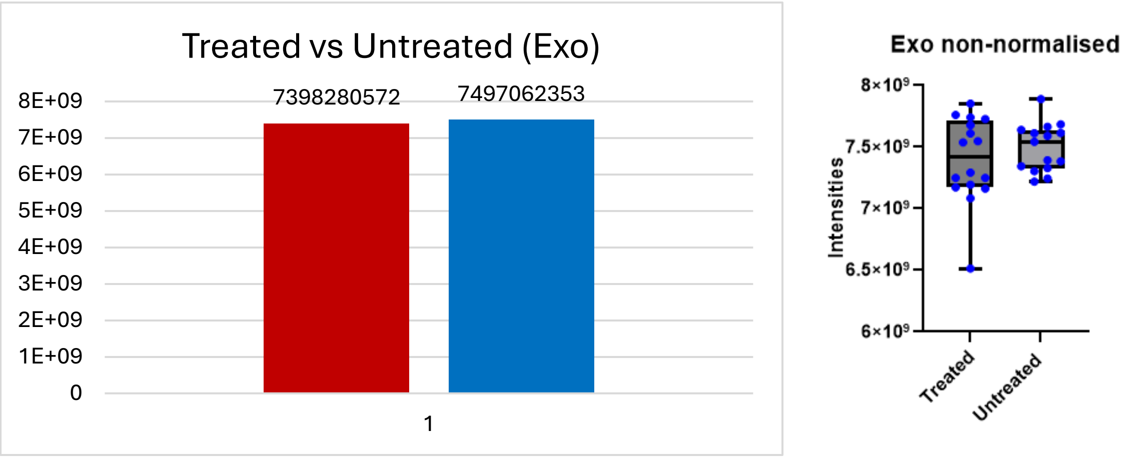

Figure S7: Total metabolite content of exo metabolome (**left**). Red: treated with 6-OHDA. Blue: untreated. Box-plot displaying the distribution of all datpoints (**right**).

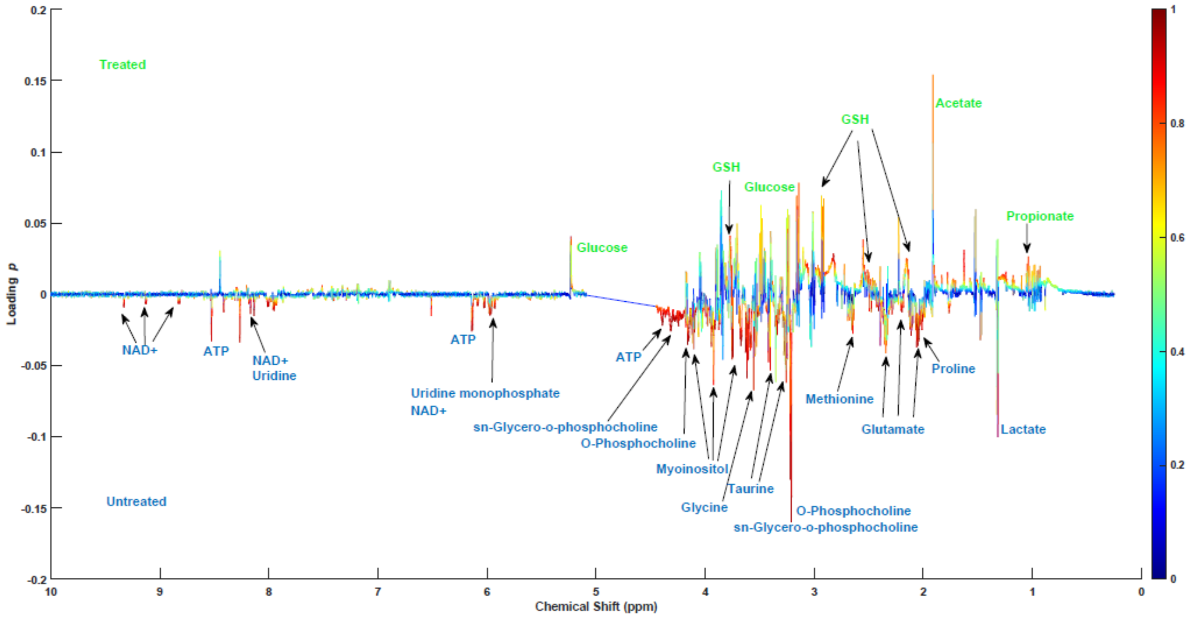

Figure S8: PCA bivariate loadings plot (PC1) of the new replicate dataset (4 biological replicates/group, 3 technical replicates) for the endo metabolome of 6-OHDA treated and untreated cells. The underlying PCA was based on n=18 spectra (24 total minus 6 outliers) and k=9101 variables, and yielded a model with 3 PCs, R^2=0.602, and Q^2= 0.311. The x-axis represents chemical shifts of metabolites in parts per million (ppm) scale. The y-axis represents loading p-values. Overlayed are the absolute values of $|p(corr)|$ as heatmap ranging from 0 (blue) being not correlated to 1 (red) being highly correlated.

Table S1: Endo metabolites identified

Decrease (6-OHDA Treated)		Increase (6-OHDA Treated)		No change (6-OHDA Treated)	
Metabolite	δ_H	Metabolite	δ_H	Metabolite	δ_H
Creatine	3.9, 3.0	Glutathione	4.56, 3.77	Alanine	3.78, 1.47
Creatine phosphate	3.9, 3.0	Acetate	1.91	Threonine	4.25, 3.59, 1.31
Creatinine	4.0, 3.0	Formate	8.45	Valine	3.61, 2.26, 1.03, 0.98
o-Phosphocholine	4.16, 3.58, 3.21	Propionate	2.17, 1.05	Isoleucine	1.23, 1.00, 0.93
Proline	4.15, 3.39, 2.35, 2.04	Ethanol	1.16	Leucine	1.68, 0.95
Lactate	4.10, 1.32	Glucose	5.23, 4.65, 3.90, 3.40	Tyrosine	7.19, 6.90
Glutamate	3.75, 2.34, 2.12, 2.04			Glutamine	2.44, 2.13
UMP	8.11, 6.01, 4.42, 4.00			Pyruvate	2.46
myo-Inositol	4.06, 3.61, 3.53, 3.29			sn-Glycero-3-phosphocholine	4.32, 3.68, 3.67
Glycine	3.55			Phenylalanine	7.42, 7.37, 7.32
NAD+	9.32, 9.12, 8.82, 8.41, 8.17,8.19, 6.08, 6.02			Succinate	2.41
Taurine	3.41, 3.26			Citrate	2.66, 2.52
Methionine	3.86, 2.16, 2.14			Isocitrate	4.02, 2.97
Glycine	3.56			2-oxoglutarate	3.00, 2.45
				Oxaloacetate	2.38

Table S2: Exo metabolites identified

Decrease (6-OHDA Treated)		Increase (6-OHDA Treated)		No change (6-OHDA Treated)	
**Metabolite**	δ_H	**Metabolite**	δ_H	δ_H	**Metabolite**
Lactate	4.11, 1.32	Leucine	1.68, 0.95	Isoleucine	1.23, 1.00, 0.93
Pyroglutamyl alanine	4.17, 4.01, 2.32, 2.11, 1.96, 1.52	Acetate	1.91	Valine	3.61, 2.26, 1.03, 0.98
Pyruvate	2.46	Alanine	3.78, 1.47	myo-Inositol	4.06, 3.61, 3.53, 3.29
Glutamate	3.75, 2.34, 2.12, 2.04	Formate	8.45	Phenylalanine	7.42, 7.37, 7.32
Taurine	3.41, 3.26	Glutamine	2.44, 2.13	Glucose	5.23, 4.64, 3.24
		Ethanol	1.16	Glycine	3.55
				τ-Methylhistidine	7.80, 7.06
				Tyrosine	6.89, 7.19
				Threonine	4.24, 3.58, 1.32
